# Wood Ear Mushrooms (*Auricularia* spp.): From Biology and Cultivation to Nutritional Value and Future Opportunities

**DOI:** 10.3390/life16101730

**Published:** 2026-10-15

**Authors:** Kritsana Jatuwong, Worawoot Aiduang, Alviti Kankanamalage Hasith Priyashantha, Didsanutda Gonkhom, Orlavanh Xayyavong, Jutamart Monkai, Shihui Wang, Saisamorn Lumyong

**Affiliations:** 1 Office of Research Administration, Chiang Mai University, Chiang Mai 50200, Thailand; kritsana.ja@cmu.ac.th (K.J.); worawoot.aiduang@cmu.ac.th (W.A.); jutamart.mon@cmu.ac.th (J.M.); 2 Department of Biology, Faculty of Science, Chiang Mai University, Chiang Mai 50200, Thailand; priyashanthahasith@gmail.com (A.K.H.P.); didsanutda8725@gmail.com (D.G.); xorlavanh@yahoo.com (O.X.); wangshihui.mocha@gmail.com (S.W.); 3 Department of Biology, Faculty of Science, Champasack University, Pakse 16010, Laos; 4 Center of Excellence in Microbial Diversity and Sustainable Utilization, Chiang Mai University, Chiang Mai 50200, Thailand; 5 Academy of Science, The Royal Society of Thailand, Bangkok 10300, Thailand

**Keywords:** agricultural wastes, mushroom cultivation, medicinal mushrooms, sustainability, wood ear mushroom, SDG2

## Abstract

Species of the genus *Auricularia* (wood ear mushrooms) are among the most economically important edible and medicinal mushrooms worldwide, particularly in Asia. Although numerous studies have investigated their taxonomy, cultivation practices, and bioactive properties, the available information remains dispersed, with limited comprehensive overviews from biological characteristics to sustainable production practices. This review summarizes and critically discusses the current knowledge of *Auricularia* species from the wild to their cultivation and production systems, including growth requirements, cultivation methods, nutritional value, medicinal potential, and future development, focusing mainly on recent advances in cultivation systems. The literature shows considerable differences among *Auricularia* species, which have significantly different environmental needs for mycelial growth and fruiting body production, indicating the importance of species-specific cultivation practices. Recent improvements in substrate formulation, spawn production, and environmental management have greatly enhanced cultivation efficiency. In addition, the use of agricultural and forestry residues as cultivation substrates supports waste recycling and contributes to the development of a circular bioeconomy. *Auricularia* species are also recognized for their considerable nutritional and medicinal value due to their high dietary fiber content and diverse bioactive compounds. This review further discusses the major pests and diseases affecting *Auricularia* cultivation, along with current management strategies, and identifies key knowledge gaps and future research priorities to improve sustainable production and commercial development.

## 1. Introduction

Mushroom cultivation has attracted increasing attention due to the rising consumer demand for nutritious, sustainable, and flavorful foods [1,2]. Among the numerous mushroom species cultivated worldwide, species in the genus *Auricularia* (wood ear mushrooms) are particularly important due to their long history of consumption, commercial value, adaptability to different environments, and recognized nutritional and medicinal properties.

The genus *Auricularia* belongs to the family Auriculariaceae. *Auricularia* is commonly known as wood ear, jelly ear, or Jew’s ear mushroom, and is sometimes called black fungus, reflecting the characteristic ear-shaped fruiting bodies, jelly-like textures, and color [3,4]. In nature, *Auricularia* species are primarily grown on dead or decaying woody materials, where they contribute to lignocellulose decomposition and nutrient cycling within forest ecosystems [5,6]. As edible mushrooms, *Auricularia* species have been used for culinary purposes in various parts of the world. In addition to their culinary value, wood ear mushrooms have a long history of use in traditional Chinese medicine because they are nutrient-dense, rich in vitamins, minerals, fiber, and protein, while being low in fat and calories. Furthermore, *Auricularia* species contain numerous bioactive compounds that have been associated with potential health-promoting effects [7,8,9,10].

Historically, *Auricularia* were primarily harvested from natural habitats for local consumption and traditional medicinal applications. Increasing market demand and concerns about the sustainability of wild harvesting have driven the development of new artificial cultivation technologies. Advances in modern cultivation practices have enabled large-scale production, offering a more sustainable alternative to wild harvesting [11]. Currently, *Auricularia* is cultivated primarily using two principal methods: traditional wood-log cultivation and substrate bag cultivation.

In Taiwan, the wood-log cultivation technique, which begins with the selection of wood logs, was traditionally used for *Auricularia* [4,12]. In contrast, bag cultivation has become increasingly popular using a synthetic medium, particularly sawdust-based formulations, which enable intensive production, shorter cultivation cycles, and improved control of environmental conditions [7,13,14,15]. The cultivation process of *Auricularia* involves several crucial steps, including species selection, mycelial growth on culture media, inoculum production (grain spawn media), substrate preparation, environmental management, and harvesting. Each of these steps plays an important role in optimizing mushroom production to achieve maximum yield and quality [15,16,17].

Despite significant progress in *Auricularia* cultivation research, knowledge remains dispersed across multiple research areas. Previous studies have primarily focused on individual aspects, such as species identification and taxonomy, mycelial growth characteristics, substrate composition, cultivation parameters, and nutritional and medicinal properties. However, relatively few studies have integrated this information into a comprehensive framework illustrating how biological characteristics, cultivation technologies, resource utilization, and sustainability considerations interact to enhance the production efficiency and product quality. Furthermore, recent developments in agricultural waste utilization, biotechnology-based cultivation strategies, and circular bioeconomy approaches have created new opportunities for improving *Auricularia* production; however, these developments have not been comprehensively reviewed.

Therefore, this review aims to provide a critical and integrated synthesis of current knowledge on *Auricularia* species. The main objectives of this review are to (i) summarize the taxonomy, diversity, ecology, and biological characteristics of *Auricularia*; (ii) evaluate progress in cultivation technologies, including culture conditions, spawn production, substrate optimization, and environmental management; (iii) summarize the utilization of agricultural and agro-industrial residues as sustainable cultivation substrates within circular bioeconomy frameworks; (iv) discuss the nutritional composition, bioactive compounds, and potential value-added applications; and (v) identify current limitations, research gaps, and future directions for improving productivity, quality, and sustainability.

## 2. *Auricularia* Species from the Wild to Cultivation

### 2.1. Taxonomy

The genus *Auricularia* was first established by the French mycologist Pierre Bulliard in 1780. In 1822, Christian Hendrik Persoon described two gelatinous species. *Auricularia mesenterica*, which was subsequently designated as the type species, and *A. sambuci*, which was later identified as a synonym of *A. auricula-judae* [18]. In 1848, Swedish mycologist Elias Magnus Fries retained *A. mesenterica* within the genus, though he established the new genus Hirneola for most other species, based on morphological differences in their fruiting bodies [19]. Although some authors continued to recognize this division into two genera until at least the 1960s [20], *Hirneola* was subsequently accepted as a synonym of *Auricularia* in 1952, according to the monograph of the genus by American mycologist Bernard Lowy [21]. However, molecular analysis based on DNA sequence data has shown that *Auricularia* (which includes *Hirneola*) forms a natural, monophyletic grouping. It has been indicated that several species have a more limited distribution than previously known, leading to the identification of further new classifications [22,23,24]. The genus *Auricularia* Bull. (Auriculariaceae, Auriculariales), with *A. mesenterica* (Dicks.) Pers. as the type species [25], currently comprises 37 species recorded globally based on morphological and modern molecular data. A comprehensive taxonomic study analyzed these species using morphological examinations and multi-gene phylogenetic analyses of 277 samples from 35 countries in Asia, Europe, North and South America, Africa, and Oceania [26]. The two most commonly cultivated species worldwide were previously named *A. auricula-judae* and *A. polytricha* [27]. However, Wu et al. [23] demonstrated through taxonomic revision that populations identified as *A. auricula-judae* from China and other parts of Asia are distinct from those occurring in Europe. Consequently, the Asian populations were reclassified as *A. heimuer*. Similarly, based on morphological characteristics and phylogenetic analyses of *Auricularia* specimens, Wu et al. [26] revised the taxonomic status of *A. polytricha* and recognized it as *A. cornea*. Therefore, further studies are required to confirm the taxonomic identity of commercially cultivated *Auricularia* strains in other countries.

### 2.2. Habitat, Ecology and Distribution

Species of the genus *Auricularia* are widely distributed throughout tropical, subtropical, and temperate regions, with their distribution influenced by climatic conditions and the availability of suitable lignocellulosic substrates [5,7,15,17]. These mushrooms are naturally found in forests, woodlands, and areas with an abundance of woody waste or residue, as they often grow on dead or decaying wood substrates, particularly on dead branches, logs, and stumps of various types of tree species [22,24,28]. The ecological association of *Auricularia* with woody substrates varies among species. Regardless of their host specificity, *Auricularia* species play an important role in the decomposition of lignocellulosic materials. As the primary decomposers, they convert deceased organic tissues into the essential nutrients required for their growth. Their mycelia secrete digestive enzymes that break down lignocellulose into smaller molecules, which are then absorbed (as nutrients). Additionally, in the process of obtaining growth nutrients from decaying organic matter, they break down the complex organic matter, contributing to nutrient cycling in forest ecosystems [29].

**Figure 1:**
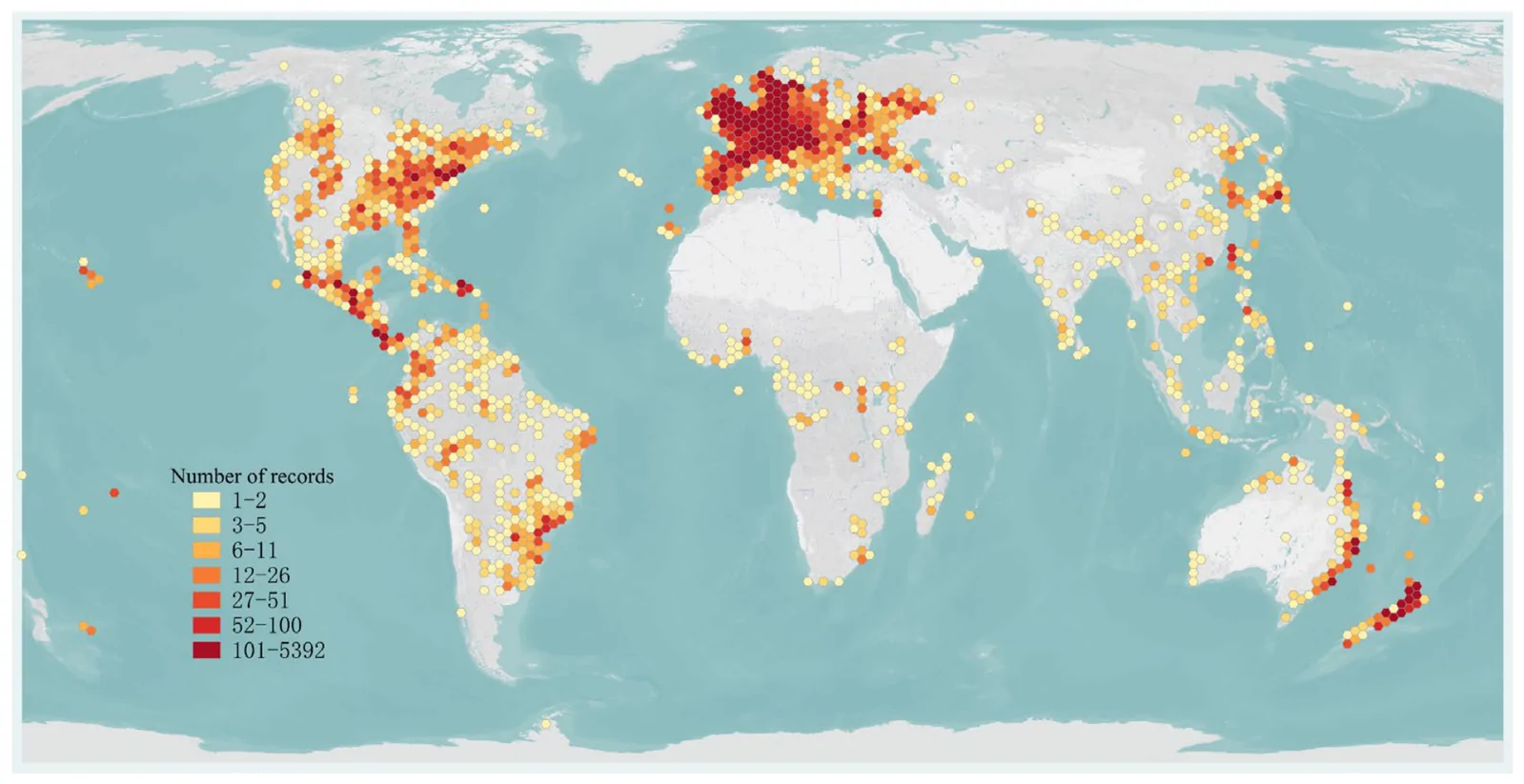
Global distribution of *Auricularia* based on occurrence records from the Global Biodiversity Information Facility (GBIF). Occurrence data were downloaded from GBIF on 10 August 2026 (161,615 records from 724 published datasets; GBIF.org, 2026; Available online: https://doi.org/10.15468/dL.khfmp8 (accessed on 10 August 2026)), of which 149,756 georeferenced records were retained for spatial analysis. These records were aggregated into 200 km hexagonal grid cells, with colors representing the number of occurrence records per grid cell from low (light yellow) to high (dark red). The map therefore represents the spatial distribution and density of available GBIF occurrence records rather than species richness or the true abundance of *Auricularia*.

Geographically, *Auricularia* has a broad global distribution across Europe, Asia, Africa, Australia, Oceania, and the Americas (Figure 1) [17,26], although the distribution ranges differ considerably among species. *Auricularia auricula-judae* has one of the broadest reported distributions, occurring primarily in temperate regions of Europe and Asia, as well as in other areas offering suitable woody substrates and favorable climatic conditions [30,31]. In tropical and subtropical regions, species such as *A. cornea*, *A. delicata*, *A. fuscosuccinea*, and *A. polytricha* have been reported from various parts of Asia, Australia, and parts of the Americas [22,24]. A global assessment based on specimens from 35 countries identified 37 species and demonstrated considerable variation in geographic distribution among species and species complexes [24]. Overall, other than climate, the distribution of *Auricularia* species is influenced by factors such as the availability of suitable wood substrates and favorable ecological conditions [5,24,32].

### 2.3. Life Cycle

**Figure 2:**
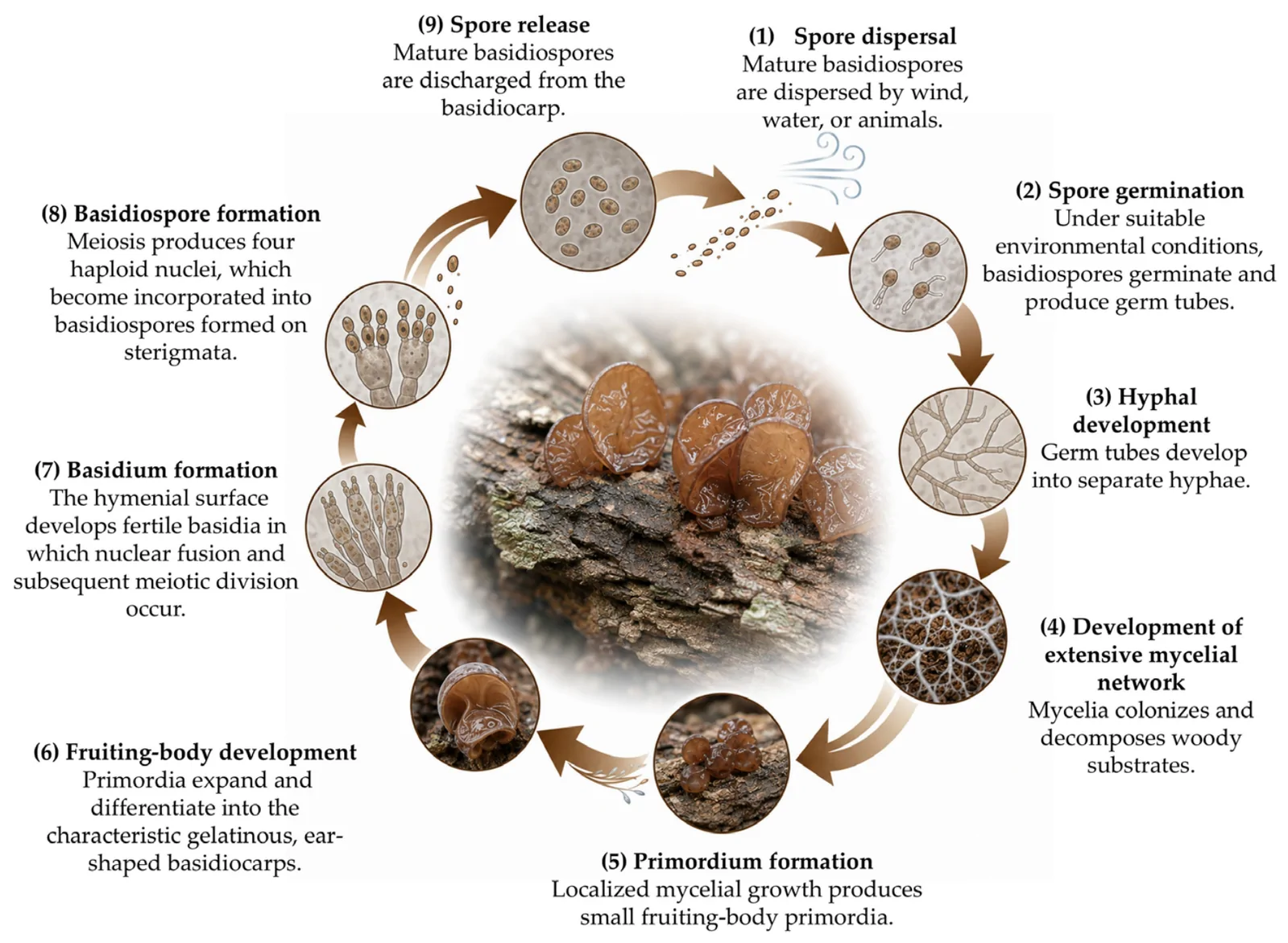
Life cycle of *Auricularia* species, depicting basidiospore dispersal and germination, hyphal and mycelial development, basidiocarp differentiation, basidium formation and meiosis, followed by basidiospore development and release.

Like many other mushrooms, the life cycle of *Auricularia* species also begins with the dispersal of microscopic spores, typically facilitated by wind, water, or animals. After reaching a suitable habitat, these spores begin to grow and develop into thin, thread-like structures called hyphae. As hyphae grow and branch, they form an extensive interconnected network-mycelium. The mycelium penetrates its host material, such as dead wood, and acquires nutrients by secreting enzymes that break down organic matter. When the mycelium matures, it reaches a stage when several environmental factors, including temperature, humidity, and nutrient availability, stimulate the formation of specialized structures known as fruiting bodies. These fruiting bodies are the distinctive ear-shaped mushrooms that emerge from the substrate. Within these fruiting bodies, spore-producing structures called basidia form and complete the process of meiosis to produce spores. Ultimately, once the fruiting bodies mature, they release spores into the surrounding environment, where they disperse to initiate a new life cycle (Figure 2) [27].

### 2.4. Biological Characteristics

**Figure 3:**
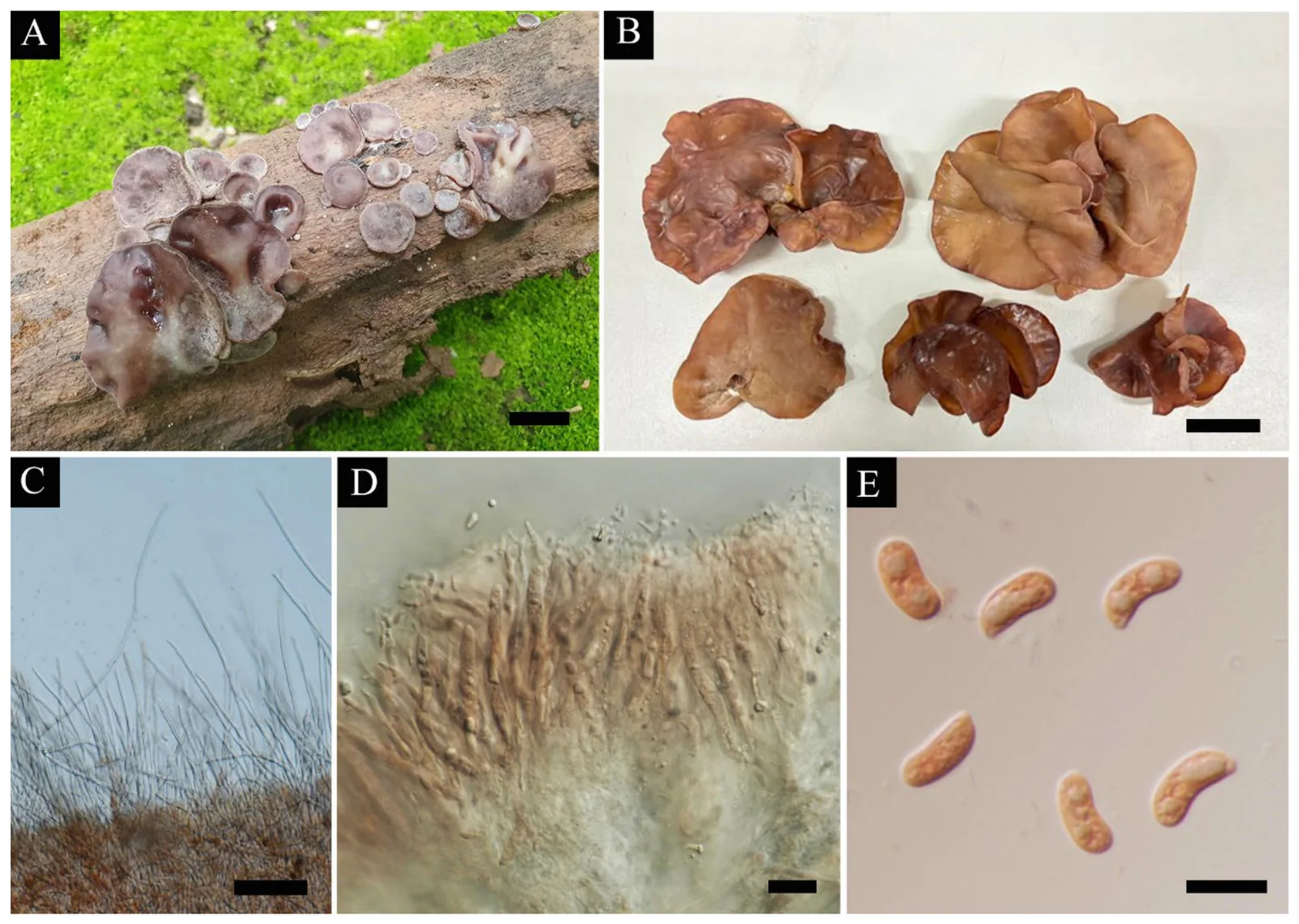
Basidiomata and microscopic structures of the *Auricularia* mushroom. (**A**) and (**B**) Basidiomata of the *Auricularia* mushroom, (**C**) Abhymenial hairs, (**D**) Basidia and (**E**) Basidiospores. Scale bars: (**A**) and (**B**) 2 cm, (**C**) 100 µm, (**D**) and (**E**) 10 µm. Photo credits: Jutamart Monkai.

Species of *Auricularia* exhibit distinct morphological traits that differentiate them from other edible mushrooms. Accurately identifying and classifying these characteristics is crucial for precise taxonomic placement within the genus. The fruiting of these species is characterized by the presence of basidiocarps that are either ear-shaped or irregularly cup-shaped [28]. Fresh basidiocarps are characterized by a gelatinous texture, attaching to the substrate either by a short lateral stipe or directly through a broad base [27,33]. The basidiocarps or fruiting bodies typically range from 3 to 8 cm; however, they can reach a maximum size of 12 cm [27]. The upper surface of the basidiocarps, known as the pileus, is typically smooth, undulating, or irregularly wrinkled, with colors ranging from shades of brown, reddish brown, to grayish brown with hair [27,34]. Microscopically, *Auricularia* is characterized by allantoid basidiospores that are cylindrical to ellipsoid in shape, smooth, and hyaline. The basidia are cylindrical to clavate and transversely three-septate with oil guttules, hyaline, and thin-walled, typically bearing four slender sterigmata to which the basidiospores are attached [24,35,36,37,38,39] (Figure 3).

## 3. *Auricularia* Cultivation

**Figure 4:**
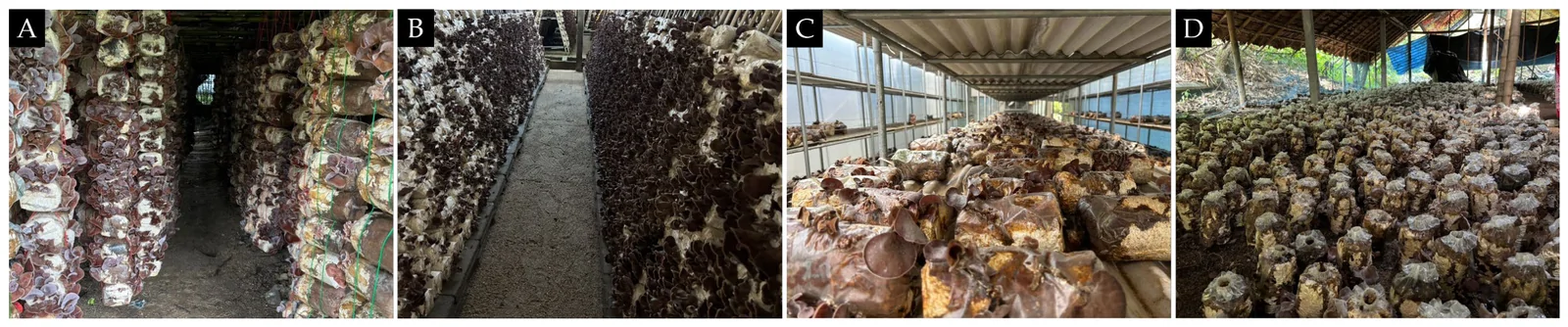
Diverse cultivation practices of *Auricularia* mushrooms across regions and production systems: (**A**) hanging bag cultivation with strings, (**B**) stacked spawn cultivation, (**C**) shelf-based cultivation, and (**D**) ground cultivation on sand. Photo credits: Worawoot Aiduang.

The cultivation of *Auricularia* has over a thousand years of history (specifically *A. auricula-judae*), particularly in China, where the mushroom was traditionally harvested from the wild prior to widespread cultivation [40,41]. It was used not only for culinary purposes but also in traditional Chinese medicine, valued for its purported health benefits [10,42]. Artificial cultivation methods for *Auricularia* were first developed in the 7th century, utilizing natural substrates such as wood logs [4,12,27]. While log cultivation is effective, after inoculation, it is time-consuming for the mushroom to produce fruiting bodies and is less controllable compared to other methods [27,40]. To address these limitations, the cultivation of *Auricularia* has been modified in recent decades, primarily through the adoption of modern techniques [43]. One of the most significant developments is the increasing use of sterilized alternative substrates such as sawdust or wood chips, which provide a more controlled environment and faster periods of growth [4,27,44]. This technique has changed *Auricularia* production, resulting in more consistent yields and effective rendering, despite the differences in cultivation practices (Figure 4), resulting in the widening of the global market. However, successful cultivation is a complicated process influenced by several interrelated factors, such as biological factors (e.g., strain selection, spawn quality, and substrate composition), aforesaid environmental factors (e.g., temperature, humidity, and substrate pH level), and management-related factors (e.g., watering, contamination control, and harvesting), each of which plays a critical role in the mushroom’s growth and development [45,46].

### 3.1. Utilization of Various Wastes as Substrates for Wood Ear Mushroom

**Figure 5:**
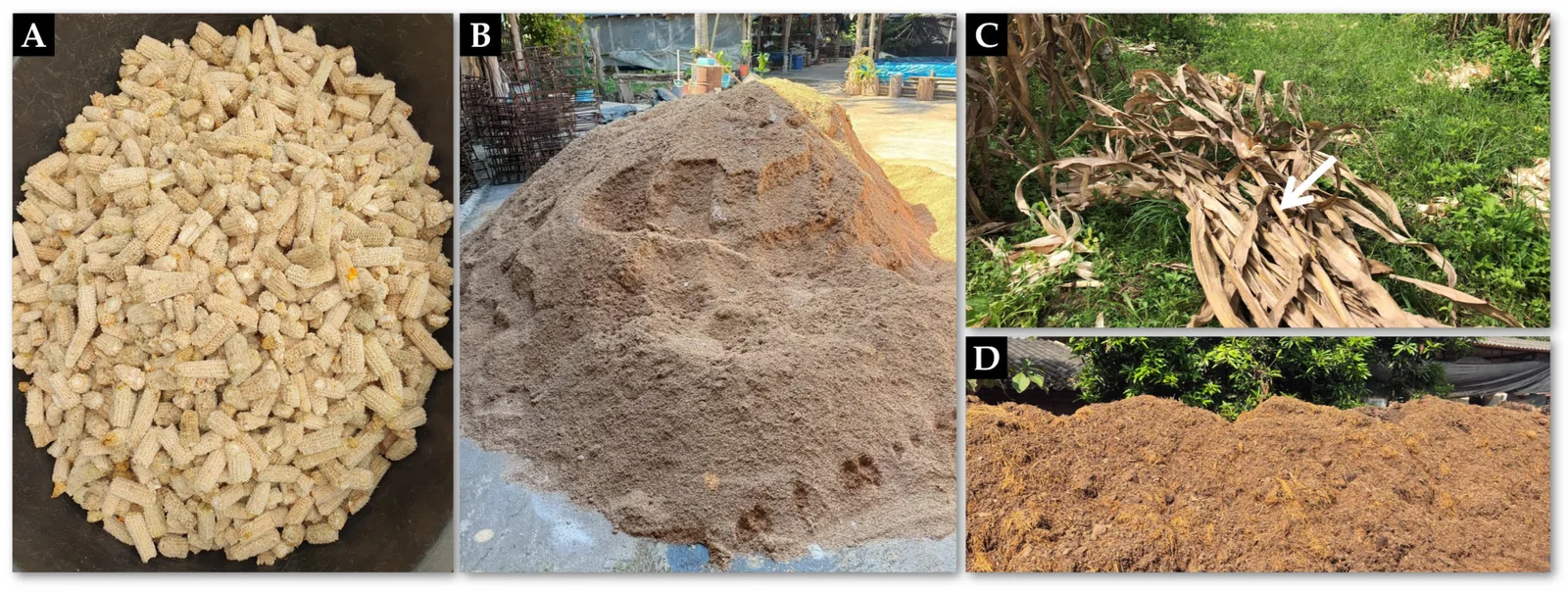
Agricultural wastes used as substrates for wood ear mushroom cultivation: (**A**) corn cobs, (**B**) sawdust, (**C**) corn stalk (shown by the arrow), and (**D**) rice straw. Photo credits: Kritsana Jatuwong.

The cultivation of *Auricularia* requires specific environmental conditions and suitable substrates that can support their growth and the highest possible production [4,47]. The selection of suitable substrates is crucial in *Auricularia* cultivation as it directly influences the growth rate, the quality of the yield, the efficiency of the cultivation process, the nutritional content of the mushrooms, as well as the overall sustainability of the production system. Substrates such as hardwood sawdust, agricultural wastes, and other organic materials rich in lignocellulosic components serve as a source of essential nutrients (e.g., carbon, nitrogen, and minerals) and appropriate physicochemical conditions (e.g., moisture content, aeration, and pH levels) required for mycelial growth [17,47,48]. Moreover, substrate bioconversion efficiency in *Auricularia* is fundamentally regulated by the initial carbon-to-nitrogen (C:N) ratio, substrate porosity, and the lignocellulose composition, particularly the ratio of cellulose, hemicellulose, and lignin [13]. Despite this, the use of agricultural waste as a substrate for wood ear mushroom has increased in popularity due to its sustainability and cost-effectiveness. Additionally, utilizing agricultural waste decreases the impact on the environment by recycling materials that would otherwise contribute to pollution [49,50]. Previously highlighted agricultural wastes (Figure 5), including rice straw, wheat straw, corn wastes, and sugarcane bagasse, are readily available in various regions and can be converted into valuable wood ear mushroom growth substrates [48,51,52,53].

Several previous studies have reported the use of various agricultural and lignocellulosic waste as substrates for cultivating different *Auricularia* species (Table 1). For instance, Ediriweera et al. [51] investigated the comparative yield of *A. polytricha* cultivated on different lignocellulosic substrates, including banana leaves, coconut leaves, coir dust, paddy straw, and sawdust. Among these substrates, banana leaves produced the highest yield (25.0 g), followed by coconut coir dust (11.1 g) and paddy straw (9.9 g). A study conducted by Onyango et al. [48] reported that maize cob supplemented with rice and wheat bran was the most suitable substrate for cultivating the Kenyan native *A. auricula*, producing the highest yield of 262.4–266.2 g and BE of 63.7–67.0%. Their study further evidenced that rather than the utilization of individual waste materials, combinations of different lignocellulosic substrates and nutritional supplements could improve mushroom production. Other than rice and wheat bran, supplements such as soybean meal, CaCO_3_ (calcium carbonate), and CaSO_4_ (calcium sulfate) have been proven to enhance the nutritional composition and physicochemical properties of the substrate, thereby promoting mycelial growth and fruiting body development.

Various studies have evaluated different substrate combinations for the cultivation of *Auricularia* species. Relatively high yields have been reported from sengon sawdust supplemented with bran, corncob, and maize straw, with yields of 314.0, 350.9, and 423.0 g, respectively. In contrast, lower yields were obtained when sengon sawdust was supplemented with rice bran and zeolite (12.4 g), coir dust (11.1 g), and paddy straw (9.9 g). Among the *Auricularia* species reported, *A. cornea* cultivated on a mixture of maize straw and oak sawdust demonstrated particularly high productivity, with yields ranging from 196.9 to 521.3 g and BE of 79.0–130.3%. Similarly, *A. heimuer* cultivated on corncob supplemented with wheat bran, soybean meal, lime, and gypsum produced a yield of 350.9 g and a BE of 70.0%. These results demonstrate the importance of substrate selection and formulation in determining the productivity of *Auricularia* and demonstrate the potential of appropriately supplemented agricultural residues as sustainable alternatives to conventional sawdust-based substrates.

As shown in Table 1, *A. cornea* produced higher yields and BE than the other evaluated species. It performed particularly well on individual waste substrates like maize straw and on mixed waste substrates, achieving yields of 196.9–521.3 g and a BE of 79.0–130.3%. The relatively high productivity observed on mixed substrates may be attributed to their more balanced C/N ratios and complementary nutrient composition, which can provide more favorable conditions for mycelial colonization and fruiting body development than individual substrates. Nevertheless, maize straw used alone also supported relatively high productivity, indicating that its suitability extends beyond its chemical composition. As a cultivation substrate, maize straw can provide nutrients, allow oxygen transfer and heat dispersion, and promote mushroom growth [54].

**Table 1:** Effects of different lignocellulosic waste substrates and nutrient supplementation on fruiting body yield and biological efficiency of cultivated *Auricularia* species.

Species	Waste Materials	Nutrient Supplementation	Moisture Content (%)	Yield (g)	Biological Efficiency (BE; %)	References
*A. auricula-judae*	maize cob	rice and wheat bran	-	262.4–266.2	63.7–67.0	[48]
	wheat straw		-	180.0–223.8	40.8–55.2	
	grass straw		-	96.4–125.6	18.2–23.8	
	sugarcane bagasse		-	117.5–136.6	25.3–30.3	
	sengon sawdust	bran, CaSO_4_, and CaCO_3_	-	314.0	66.2	[55]
	reed	rice bran and zeolite	-	17.9	32.4	[56]
	sengon sawdust		-	12.4	23.6	
	oak sawdust	wheat bran	-	17.0	-	[57]
	oak sawdust: poplar sawdust: cotton seed meal		-	24.0	-	
	miscellaneous sawdust: walnut sawdust (1:3)	wheat bran, lime, and gypsum	60	-	-	[58]
*A. cornea*	rubber sawdust	rice bran, spent brewery grain, glutinous rice flour, pumice sulfate and CaCO_3_	70	19.4–76.0	22.8	[15,17]
	maize straw	wheat bran, CaCO_3_, and CaSO_4_	60	423.0	105.7	[54]
	maize straw: fine oak sawdust: coarse oak sawdust			196.9–521.3	79.0–130.3	
	beech sawdust	CaCO_3_	70	68.7	27.9	[52]
	hornbeam sawdust			71.7	29.1	
	wheat bran			56.7	23.1	
	rice bran			61.7	25.1	
	beech sawdust/hornbeam sawdust mixed with wheat bran or rice bran			87.7–131.7	35.5–53.1	
	sawdust			-	95.3–148.1	
*A. heimuer*	corncob	wheat bran, soybean meal, lime, and gypsum	60	350.9	70.0	[59]
	bamboo sawdust	wheat bran, glucose, gypsum, and lime	-	-	-	[60]
*A. polytricha*	sawdust	-	-	32.0–65.4	5.6–15.6	[61]
	banana leaves	rice bran, CaCO_3_ and MgSO_4_	-	25.1	-	[51]
	coir dust		-	11.1	-	
	paddy straw		-	9.95	-	
	Sawdust with or without oil palm waste	rice bran, spent grain and spent yeast	65–85	8.1–63.1	25.2–288.9	[13]
	rice straw: coconut sawdust	-	-	14.2–75.6	2.8	[53]
	sawdust mixed with *Panicum repens* stalk/*Pennisetum purpureum* stalk/*Zea mays* stalk	rice bran and CaCO_3_	65	-	-	[14]
	good lumber sawdust	rice bran and lime	-	254.0	30.8	[62]
*A. thailandica*	oak sawdust: rice bran	CaSO_4_	65	60.8	17.1	[7]

**Note:** Chemical compounds are abbreviated as follows: CaSO_4_ = calcium sulfate, CaCO_3_ = calcium carbonate, and MgSO_4_ = magnesium sulfate.

### 3.2. Cultivation Practices for Auricularia spp.

**Figure 6:**
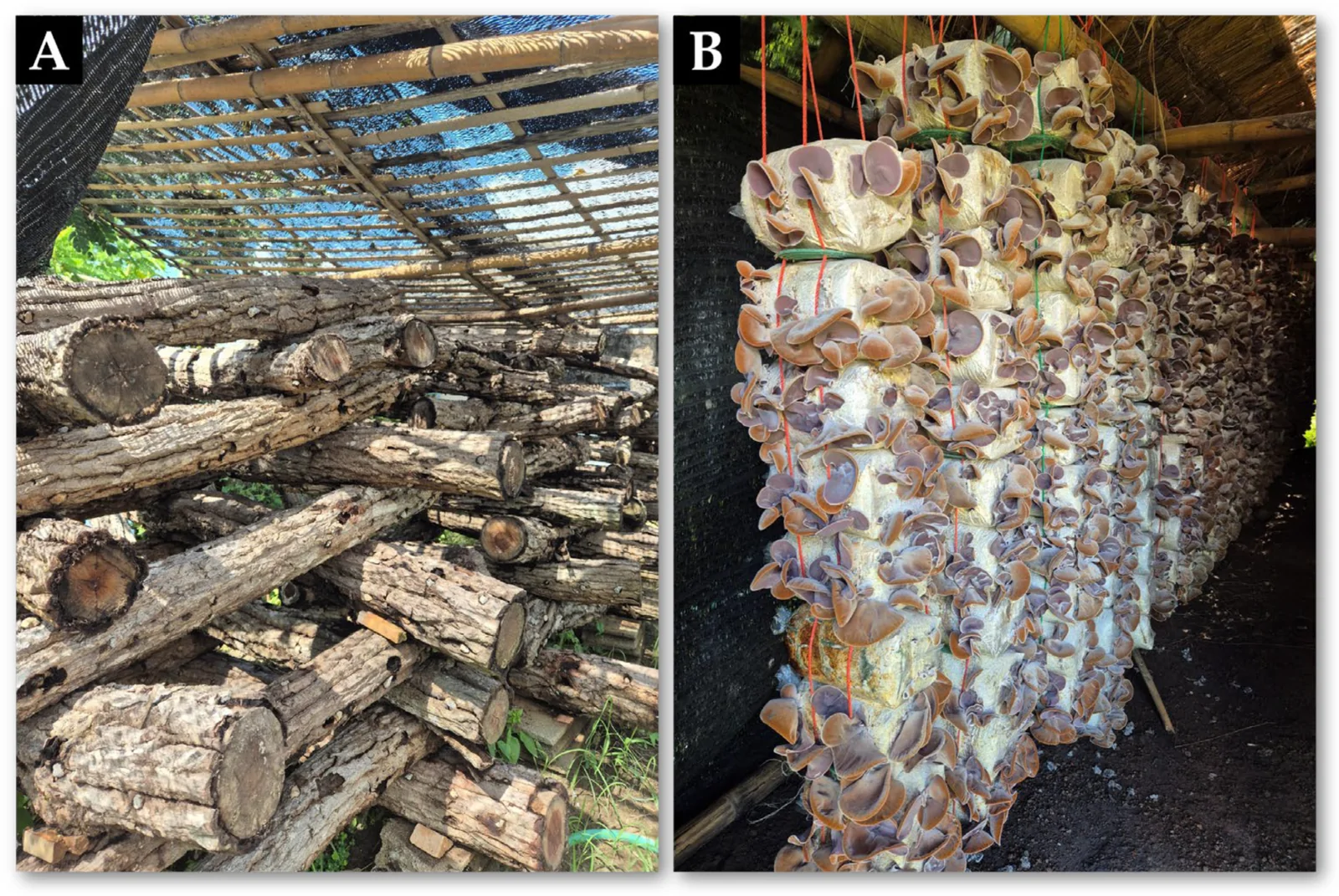
Cultivation practices of *Auricularia* spp.: (**A**) log cultivation and (**B**) plastic-bags cultivation. Photo credits: Kritsana Jatuwong.

Traditionally, *Auricularia* mushrooms were grown using log cultivation (Figure 6A), a method that closely resembles their natural growth conditions. However, cultivation using agricultural-waste-based substrates packed in plastic bags (Figure 6B) has become increasingly common because it can shorten the production cycle, facilitate management, and provide more consistent and higher-quality yields than traditional log cultivation, while also reducing dependence on natural forests and promoting sustainable industry development [17,41,60]. Modern *Auricularia* production generally relies on lignocellulosic agricultural residues as cultivation substrates as well as on controlled moisture content and environmental conditions [17,41,62]. Regis and Geösel [4] reported temperature ranges of 24–30 °C during spawn running, 12–20 °C for primordium formation, and 21–30 °C during fruiting body development. The corresponding relative humidity requirements were 90–95%, 90–100%, and 85–90%, respectively.

### 3.3. Types of Spawn Inoculum

#### 3.3.1. Grain Spawn

**Figure 7:**
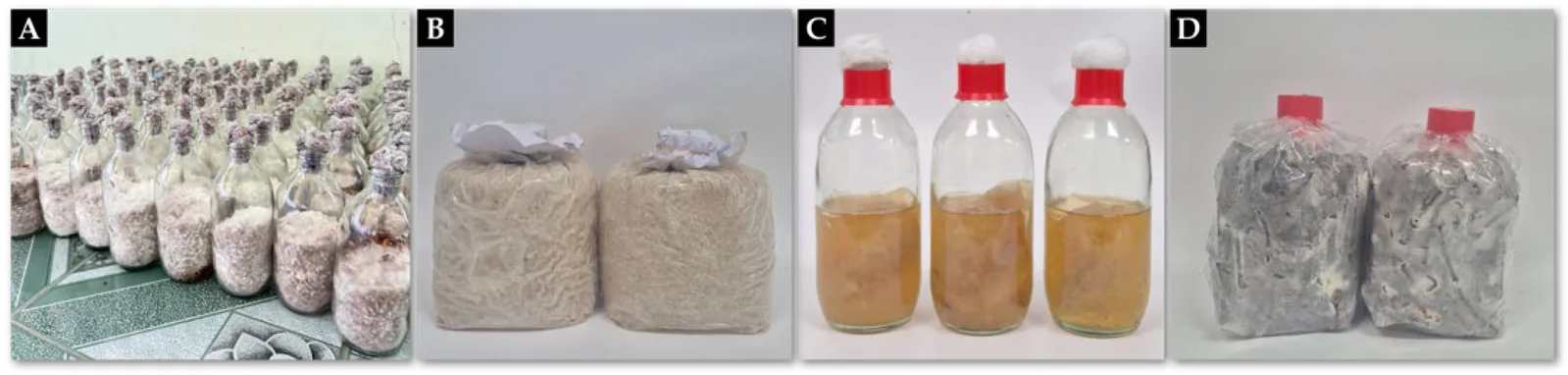
Types of spawn inoculum used for *Auricularia* species: (**A**) grain spawn, (**B**) sawdust spawn, (**C**) liquid spawn and (**D**) stick spawn. Photo credit: Kritsana Jatuwong.

Grain spawn (Figure 7A) is commonly used as an intermediary inoculum in the production process of *Auricularia* species. Various cereal grains such as paddy, maize, wheat, millet, sorghum, and rye have been evaluated as carrier materials for spawn preparation. These grains can support vigorous mycelial colonization, promoting the development of a dense mycelial network on the grain surface and abundant fluffy mycelial growth throughout the substrate [4]. However, the effectiveness of each grain type can vary depending on the *Auricularia* species and strain. Onyango et al. [63] reported that supplemented millet and sorghum grains were suitable substrates for the spawn production of *A. auricula*, promoting rapid mycelial colonization and the formation of high-quality spawn. Among the formulations evaluated, a substrate consisting of 40% millet, 40% sorghum, and 20% wheat bran exhibited the fastest colonization rate, achieving complete mycelial growth within 10 days. The authors revealed that the enhanced colonization rate may be attributed to the optimal balance of energy-rich components (lipids) and essential nutrients—carbon and nitrogen. In addition, lipids and minerals collectively promoted vigorous mycelial development. Consistent with these findings, Walker et al. [17] evaluated several cereal grains and agricultural substrates, including paddy grain, rice straw, rye grain, sawdust, sorghum, and wheat grain for spawn production and mycelial growth of three *A. cornea* isolates (MFLUCC18-0346, MFLUCC18-0347, and MFLUCC23-0084). According to their results, complete mycelial colonization occurred 27 days after inoculation, with sorghum and paddy grains found to be the most suitable substrates, consistently supporting superior mycelial growth across all three isolates. Similarly, Zurbano [62] reported that sweet sorghum supported the fastest mycelial growth of *A. polytricha*, with colonization completed within five days, a growth rate of 16 mm/day, and dense mycelial development. Aguilar et al. [53] reported that cracked corn is a suitable spawning material for *A. polytricha*. Moreover, rye grains have been used as spawn for *A. thailandica* strain MFLU162122 [5]. Table 2 further indicated the variation in the performance of different spawn types among *Auricularia* species, highlighting the influence of spawn substrate and cultivation conditions on their cultivation performance. Notably, the selection of grain types for spawn production is typically based on their local availability and cost-effectiveness [64].

#### 3.3.2. Sawdust Spawn

Sawdust spawn (Figure 7B) is one of the most commonly used spawn types for the cultivation of *Auricularia* species. Sawdust is particularly suitable for wood-decomposing fungi, which naturally inhabit lignocellulosic substrates, and it supports vigorous mycelial development [14]. The type of hardwood and the nutritional supplements incorporated into the substrate can strongly influence mycelial growth and spawn quality [52]. Accordingly, several studies have evaluated sawdust-based substrates as inoculum sources for the cultivation of *Auricularia* species. For example, Liang et al. [14] and Wu et al. [16] reported the use of sawdust substrates supplemented with 10% rice bran and 1% calcium carbonate for cultivation of *A. polytricha.* In another study, sawdust derived from two hardwood trees, namely beech and hornbeam, and supplemented with wheat bran, rice bran, and corn flour, was evaluated for spawn production of *A. cornea*. The results showed that a substrate consisting of 70% beech sawdust and 30% wheat bran incubated at 28 °C produced the highest mycelial growth rate (9.3 mm/day) and the shortest spawn run period (9 days) [52].

#### 3.3.3. Liquid Spawn

Among the various spawn types used in *Auricularia* cultivation, liquid spawn (Figure 7C) has been explored as an alternative inoculation method, though only in a handful of studies. Liquid spawn (or submerged liquid fermentation) is typically produced in a nutrient-rich medium containing carbon and nitrogen sources to support mycelial development [64]. Liquid spawn has been investigated as an alternative inoculation method for *Auricularia* cultivation. To support mycelial growth and protein synthesis, media are typically formulated with carbon sources (e.g., potato extract, glucose, and brown sugar), nitrogen sources (e.g., peptone and wheat bran), and essential inorganic mineral salts [58,59]. In more detail, for example, a liquid medium used for *A. auricula* consisted of potato (100 g/L), wheat bran (40 g/L), glucose (20 g/L), peptone (2 g/L), brown sugar (15 g/L), MgSO_4_ (magnesium sulfate) (1 g/L), and KH_2_PO_4_ (potassium dihydrogen phosphate) (2 g/L) [58]. Similarly, liquid spawn of *A. heimuer* was prepared using a medium containing peeled potato (200 g/L), glucose (20 g/L), peptone (5 g/L), and KH_2_PO_4_ (3 g/L) [59].

#### 3.3.4. Stick Spawn

Stick spawn (Figure 7D) has been developed as an alternative method for *Auricularia* spawn production [59,67]. Various lignocellulosic materials have been investigated for stick spawn preparation, with their suitability depending on chemical composition, availability, and capacity to support mycelial growth. For *A. heimuer*, branch-based spawn prepared from *Populus* branches has been developed as a tertiary spawn material. In this method, *Populus* branches are treated with saturated lime water to improve substrate conditions and are subsequently supplemented with sawdust, bran, soybean meal, lime, and gypsum [59]. The supplemented branch substrate provides a suitable environment for mycelial development, demonstrating the potential of woody biomass materials as effective carriers for *Auricularia* spawn production. However, preparation methods and substrate formulations may vary depending on the *Auricularia* species and locally available resources.

Recently, Xayyavong et al. [67] evaluated several agricultural biomass residues, including giant mimosa stalk, rubber tree wood, maize stalk residues, and banana leaf sheath, as potential materials for stick spawn production for *A. polytricha*. The materials were preconditioned by soaking in potato dextrose broth (PDB) to achieve an appropriate moisture level for mycelial colonization. Among the tested materials, giant mimosa stalk exhibited superior performance, supporting the most extensive and dense mycelial growth. This enhanced performance was associated with its higher cellulose and hemicellulose contents, which provide accessible carbon sources for fungal growth. These findings highlight the importance of selecting lignocellulosic materials with suitable physicochemical properties to improve stick spawn quality and suggest that lignocellulosic biomass residues can serve as sustainable alternatives for *Auricularia* spawn production.

#### 3.3.5. Comparative Performance of Spawn Types

It is not clear that various spawn types have been used for the cultivation of *Auricularia* spp., with the spawn substrate and material influencing mycelial colonization, incubation period, production, and BE (Table 2). Regardless of spawn type, the inoculum was used at different rates and incubated under fruiting conditions ranging from approximately 15 to 32 °C and 64–95% relative humidity.

According to the literature, for *A. cornea*, *A. polytricha*, and *A. thailandica*, grain spawn prepared from wheat, rye, sorghum, and cracked corn was used. Under fruiting conditions of 22–32 °C and 64–95% relative humidity, incubation periods ranged from 14 to 52.9 days, while BE values ranged from 2.8 to 106.9%. Sawdust spawn has also been used for *A. cornea* and *A. polytricha*, using substrates such as beech, oak, and rubber tree sawdust. For *A. cornea*, sawdust-based spawn resulted in BE values ranging from 23.1 to 53.1% and from 67.1 to 130.3%, depending on the substrate formulation and cultivation conditions, with production yields ranging from 56.7 to 457.3 g. Liquid spawn prepared using nutrient-rich media has been reported for *A. auricula* and *A. polytricha*. For *A. auricula*, inoculation with 15 mL of liquid spawn per bag, followed by incubation at 22 °C and 90–95% relative humidity, resulted in an incubation period of approximately 40–50 days and a BE of approximately 6.0–8.0%. Stick spawn prepared from *Populus* branches or giant mimosa stalks has been used for *A. heimuer* and *A. polytricha.* For *A. heimuer*, cultivation using *Populus* branch-based stick spawn resulted in production yields of 157.7–350.9 g and BE values of 34.0–70.0%.

A study by Xayyavong et al. [67] further evaluated the cost–benefit aspects of different spawn types, comparing production costs per unit (bag and bottle) with the revenue and profit generated per mushroom production batch. According to their analysis, the production cost per unit differed substantially among the four inoculum types. Liquid spawn prepared using potato dextrose broth (PDB) had the highest production cost at USD 2.80 per bottle, followed by sorghum grain spawn at 0.29 USD per bottle, sawdust spawn at 0.24 USD per bag, and giant mimosa stalk (GMS) stick spawn at only 0.074 USD per bag. Revenue and profit were calculated based on the number of inoculated bags and prevailing mushroom prices, with profit calculated as the difference between revenue and production costs. These reports indicated that the selection of spawn type and substrates can have important economic impacts for *Auricularia* cultivation. Therefore, spawn selection may vary among countries and production systems depending on the local availability and cost of raw materials, as well as the resulting production yield and biological efficiency.

### 3.4. Media, Nutritional and Environmental Requirements for Mycelial Growth

The mycelial growth of *Auricularia* species depends on various nutritional and environmental factors, including the composition of the culture medium and incubation conditions. Although various culture media formulations can support the growth of different species, the quality and efficiency of mycelial growth depend on the nutrient composition of the medium. As summarized in Table 3, numerous studies have investigated the optimum conditions for mycelial growth in *Auricularia* species by adjusting physical and nutritional parameters, such as carbon and nitrogen sources, initial pH, and incubation temperature. The wide range of optimal conditions reported among *Auricularia* species reflects the broad physiological range within this genus.

#### 3.4.1. Culture Media

The selection of an optimal culture medium is an essential requirement for improving mycelial growth in *Auricularia* species, as the nutrients provided by the medium directly influence mycelial growth rate, mycelial density, and biomass production. Various culture media have been used for the growth of *Auricularia* species, including Czapek–Dox, glucose peptone, malt extract agar, mesangial cell medium, potato dextrose agar, yeast extract agar, yeast mannitol agar, and Leonian medium [17]. For *A. auricula-judae*, Jo et al. [47] reported that potato dextrose agar (PDA) and MCM were the most suitable media for mycelial growth based on colony diameter and mycelial density. For *A. cornea* (syn. *A. polytricha*), hornbeam and beech sawdust extract agar supported the highest mycelial growth rate, measuring 7.5 mm/day. These findings indicate that wood-derived and plant-based extract media can provide effective and potentially economical alternatives to conventional commercial agar media for the growth of *Auricularia* mycelia. In addition, Walker et al. [17] reported that rice bran sucrose agar (RSA) produced the highest dried mycelial biomass in *A. cornea*, approximately 0.10–0.17 g. Similarly, Tra et al. [68] found that two *A. cornea* strains, white and brown, demonstrated robust growth in MCM broth, producing 0.09 g and 0.10 g of dried mycelial biomass, respectively. For *A. polytricha*, responses to culture media also vary among strains. Xu and Yun [71] reported effective mycelial growth in yeast malt (YM) medium, while Zurbano [62] reported that coconut water gelatin (CWG) is the most favorable medium, resulting in the fastest mycelial ramification (6.3 days) and the highest growth rate (13.2 mm/day), with notably dense mycelial development. More recently, Aguilar et al. [53] reported that potato sucrose agar (PSA) supported the highest daily mycelial diameter in *A. polytricha*, measuring 12.8 mm/day.

#### 3.4.2. Carbon Sources

Carbon sources are critical factors influencing the mycelial growth of Basidiomycetes as they serve as primary energy sources required for fungal metabolism, cellular development, and biomass production. Various carbon sources have been evaluated for their effects on mycelial growth in *Auricularia* species; however, the optimal carbon source varies depending on species, strain characteristics, and cultivation conditions (Table 3). For *A. auricula-judae*, mannose has been reported as the most suitable carbon source for promoting mycelial growth. In *A. cornea*, the carbon source differs among strains and experimental conditions. Walker et al. [17] reported that sucrose effectively supported mycelial growth of *A. cornea* on RSA, while Sun et al. [69] found that mannitol at 1.0 g/L was the most favorable carbon source for a wild *A. cornea* strain cultured on PDA, resulting in the highest mycelial growth rate. These variations in carbon utilization among *A. cornea* strains may be associated with differences in metabolic adaptation, genetic background, geographic distribution, and the environmental conditions of their native habitats. For *A. delicata*, rhamnose was reported to be the most effective carbon source for mycelial production [70]. For *A. polytricha*, sucrose has consistently proven to be an effective carbon source across different culture media, as demonstrated by Xu and Yun [71] using YM medium and Aguilar et al. [53] using potato sucrose agar. Interestingly, *A. polytricha* is able to grow on various media formulations, suggesting broad metabolic adaptability and efficiency in utilizing different carbon sources. These findings indicate that carbon source optimization is species- and strain-dependent and should be considered an important factor in developing suitable culture media for efficient *Auricularia* mycelial production.

#### 3.4.3. Nitrogen Sources

Among other factors, the mycelial growth of *Auricularia* species depends on the availability of nitrogen sources in precise proportions. Previous studies have demonstrated that organic nitrogen sources are generally more effective than inorganic nitrogen sources in promoting mycelial development due to their higher accessibility and nutritional suitability for fungal growth [47,69,70,71]. For *A. auricula-judae*, malt extract was found to be the most suitable nitrogen source. When 2% glucose was used as the carbon source, a C/N ratio of approximately 10:1 provided suitable conditions for mycelial growth [47]. In submerged culture of *A. polytricha*, yeast extract at a concentration of 10 g/L was the most effective nitrogen source and enhanced mycelial dry weight to 6.1 g/L [71]. Similarly, yeast extract was reported as the most suitable nitrogen source for wild *A. cornea*, confirming the superior performance of organic nitrogen sources in supporting mycelial growth compared with inorganic nitrogen sources [69]. For *A. delicata*, media supplemented with beef extract and peptone produced stronger and denser mycelial growth, indicating that these organic nitrogen sources can also support its growth [70].

#### 3.4.4. pH and Temperature

pH and temperature are two critical physicochemical factors that influence mycelial growth of *Auricularia* species in both solid and submerged culture. The existing literature has demonstrated that most *Auricularia* species generally exhibit favorable mycelial growth under slightly acidic to near-neutral conditions, with optimal temperatures typically ranging from 25 to 32 °C. However, these optimal parameters vary considerably among species and strains, underscoring the need for strain-specific optimization to maximize mycelial growth (Table 3).

## 4. Environmental Factors for Fruiting Body Development

Environmental conditions play a crucial role in the initiation of primordia and the subsequent development of fruiting bodies in *Auricularia* species. The morphology and successful development of *Auricularia* basidiomata are strongly influenced by several environmental factors, including temperature, relative humidity (RH), light, carbon dioxide concentration (CO_2_), and air exchange rate. These factors provide the necessary external signals to trigger the switch from vegetative growth to reproductive development. Table 1 summarizes the optimal environmental conditions reported for commercially and ecologically important *Auricularia* species.

Temperature is one of the most influential factors affecting all stages of *Auricularia* cultivation, particularly fruiting and development. Across the genus, the optimal temperature for fruiting varies among species, generally ranging from 17 to 32 °C. *Auricularia heimuer* exhibits the narrowest optimal fruiting temperature range at 20–22 °C [59]. For other species, the reported temperature ranges for fruiting are 18–30 °C for *A. auricula*, 17–28 °C for *A. delicata*, and 22–30 °C for *A. cornea* (Table 4). Among the species reviewed, *A. polytricha* has a relatively broad fruiting temperature range of 23–32 °C (Table 4), suggesting its potential suitability for cultivation in tropical and subtropical conditions.

High relative humidity is essential for primordium initiation, differentiation, and the maintenance of normal basidiomata morphology. Most *Auricularia* species require humidity levels above 80% during fruiting to prevent shriveling of the fruiting bodies [4]. In general, fruiting bodies develop well at approximately 90–95% RH. However, previously reported optimal relative humidity varies among species, from 60% to 96%. Specifically, optimal RH ranges of 90–95% have been reported for *A. auricula*, 75–90% for *A. cornea*, 80–90% for *A. heimuer*, and 80–96% for *A. polytricha* (Table 4). In contrast, *A. delicata* exhibits a broader humidity tolerance ranging from 60 to 95%.

Light is also an important environmental factor required for fruiting body initiation and development in *Auricularia* species [4]. Several studies have shown that alternating light and dark periods can promote fruiting body formation and influence developmental characteristics. A defined photoperiod of a 12 h light/12 h dark cycle is commonly used for the cultivation of *Auricularia* species. For example, fruiting body development of *A. auricula*, *A. cornea*, and *A. delicata* has been successfully achieved under a 12 h light/dark cycle. In contrast, *A. polytricha* has been cultivated under continuous illumination (24 h light) at relatively low light intensities of 179 lux [61] and 200 lux [73]. These reports suggest that *A. polytricha* may possess greater adaptability to prolonged light exposure and may not require a strict photoperiod for fruiting induction when adequate light intensity is provided. However, the specific light requirements may vary among *Auricularia* species, indicating the importance of optimizing photoperiod and light intensity for efficient commercial cultivation.

In addition to temperature, humidity, and light, adequate air exchange and reduced CO_2_ concentration are important during fruiting body development. Most importantly, CO_2_ is a critical environmental factor influencing the growth and morphogenesis of Basidiomycetes. High CO_2_ concentrations generally suppress primordium formation and may result in abnormal basidiomata development, particularly during fruiting body formation [4,65]. Regis and Geösel [4] emphasized that CO_2_ concentrations for *Auricularia* spp. differ among cultivation stages, with levels of >5000–20,000 ppm during spawn run, 600–1000 ppm during primordium formation, and 2000–5000 ppm during fruiting body development. Under field conditions, Drewinski et al. [65] reported an average CO_2_ concentration of 1057 ppm, with values ranging from 684 to 2297 ppm, during the cultivation of *Auricularia cornea*. Therefore, proper ventilation is necessary to maintain suitable CO_2_ conditions and promote normal fruiting body development.

## 5. Postharvest Technologies, Diseases, Pests and Management Strategies

### 5.1. Postharvest Handling

Like other cultivated mushrooms, harvesting and postharvest management of *Auricularia* spp. directly influence product quality and storage stability. The optimal harvest stage is generally characterized by fully developed and expanded basidiocarps with a characteristic gelatinous texture and uniform pigmentation. Mature *A. auricula* fruiting bodies have been described as dark brown, gelatinous, and flaky, with a typical diameter of 15–35 mm and a thickness of 0.7–1.0 mm [75]. Delayed harvesting may result in deterioration of *Auricularia* fruiting bodies, including excessive expansion and morphological changes that can reduce product quality [75]. Postharvest processing commonly involves dehydration to reduce the high moisture content of fresh fruiting bodies and extend storage stability. Fresh *Auricularia* fruiting bodies, such as *A. auricula*, contain approximately 90% moisture and, if they are not harvested quickly, may be highly susceptible to decay and deterioration due to their high susceptibility to microbial growth. The most common drying methods for preserving mushrooms include natural drying and hot-air drying, which can be carried out in an extensive temperature range (37.8–82.2 °C) [76,77]. Zhu et al. [76] further reported that heat-pump drying of *A. auricula* at 50 °C resulted in the highest rehydration ratio, indicating favorable rehydration performance after drying.

Rehydration of dried mushrooms is typically achieved by soaking the dried material in water at temperatures ranging from ambient to high temperatures. During this process, the mushroom tissue can reabsorb water and regain its original structure. Ásványi et al. [77] found that dried mushrooms recovered texture profile, including hardness, springiness, chewiness, gumminess, and cohesiveness, comparable to fresh mushrooms after a 20 min soak at 40 °C.

For long-term storage of *Auricularia* spp., postharvest technologies, such as nano-edible coatings, packaging type, and storage temperature, play important roles in preserving the mushroom’s physicochemical and antioxidant quality. Kusumiyati et al. [78] reported that a combination of a sodium alginate-based nano-edible coating with vacuum-sealed packaging and storage at 10 °C showed the most favorable results, maintaining moisture, total anthocyanin, tannin, total phenolic and flavonoid content and antioxidant capacity over six days of storage.

### 5.2. Diseases, Pests and Management Strategies

Fungi and mites are among the most important pests and disease-associated agents affecting *Auricularia* spp. cultivation and can cause significant crop losses. In comparison, there have been few reports of disease caused by bacteria and nematodes in *Auricularia* cultivation [79]. A summary of the major diseases and mite infestations associated with *Auricularia* spp. is presented in Table 5.

The notable increase in disease outbreaks, with new pathogens and disease records emerging at an unprecedented rate, poses severe threats to the sustainable production of *Auricularia* [80,81]. The last decade has seen the identification of novel pathogens, such as *Hypomyces cornea* and *H. mycophilus*, and the expansion of known pathogens into new geographic regions and host varieties [82]. The genetic plasticity of both *Auricularia* spp. and pathogens, as revealed by recent genomic studies, suggests a dynamic coevolutionary landscape that may facilitate the emergence of new disease threats [83,84]. Furthermore, the extensive use of artificial substrates, high-density cultivation, and suboptimal environmental conditions may promote favorable conditions for pathogen colonization and disease development, thereby increasing the risk of disease infections in commercial cultivation systems [81].

Slippery scar caused by *Scytalidium auriculariicola* and *Scytalidium lignicola* is one such disease [81,85]. As previously highlighted, the emergence of new disease records appears to be a future concern associated with *Auricularia* spp., particularly in countries such as China and Korea, where these mushrooms are produced on a large scale [86,87,88]. In China, from 2010 to 2016, cobweb disease caused by *Cladobotryum cubitense* was reported in *A. polytricha* in Fengxian County, Jiangsu Province, with the pathogen reportedly affecting more than 70% of the fruiting bodies [87]. The disease has subsequently been associated with other pathogens, particularly *Hypomyces mycophilus*. In 2021, *H. mycophilus* infection was reported in association with *A. cornea* in Fangshan District, Beijing, China, where approximately 20% of fruiting bodies were affected [83]. In the latter part of 2021, Zhang et al. [88] reported the infection of the disease in *A. heimuer* cultivated in mushroom farms in Fujian Province, China. In 2022, the disease was reported for the first time in Guizhou Province, China, where it affected *A. cornea* cultivation, with disease incidence reaching 30% [84]. During 2016 and 2022, farmers practicing traditional greenhouse cultivation in Shifang, a county-level city in Sichuan Province, China, reported brown rot caused by *Trichoderma pleuroticola* in *A. cornea*, with disease incidence reaching 20% [80]. The disease can cause serious losses if infected fruiting bodies are not immediately removed. Under greenhouse conditions, the pathogen reportedly spreads to 100% of the crop within 10–15 days [80]. *Trichoderma pleuroticola* has also been reported as a highly pathogenic fungus affecting *A*. *auricula*. In vitro studies showed that its growth rate was 3.26–3.52 times higher than that of *A*. *auricula*, allowing it to compete effectively for space and nutrients and completely inhibit the mycelial growth of *A*. *auricula* in confrontation cultures [89]. The disease can cause substantial production losses if infected fruiting bodies are not promptly removed. Under greenhouse conditions, the pathogen was reported to spread throughout the crop, reaching 100% disease incidence within 10–15 days [80]. *Trichoderma pleuroticola* has also been identified as a highly pathogenic fungus affecting *A. auricula*. In vitro studies showed that its growth rate was 3.26–3.52 times faster than that of *A. auricula*, enabling it to compete effectively for space and nutrients and completely inhibit *A. auricula* mycelial growth in confrontation cultures [89].

The mite, *Luciaphorus auriculariae,* is one of the most destructive pests that has long been known for affecting *Auricularia* spp. The damage caused by the mite is significant, e.g., 10–50% total loss of the crop reported in Fujian Province, China [90]. *Luciaphorus perniciosus* is another serious pest of mites that is recorded as affecting *Auricularia* spp., which can rapidly develop and reproduce under favorable conditions, resulting in rapid damage to *Auricularia* cultivation. Mites like *L. perniciosus* feed on the mycelium and can affect different stages of mushroom development by inhibiting mycelial growth and reducing sporophore formation. The ability of mites to spread through the air may also affect transmission between mushroom cultivation houses [91]. Mites belonging to the genus *Tyrophagus* are also commonly associated with *Auricularia* cultivation and have been reported to cause Krepes disease. Those mites can survive for about one month without any food sources under humid conditions (20–25 °C). In addition, mites are tiny and difficult to observe, especially when they occur in fungal composts or cultivation substrates, thus making them difficult to detect and control [92,93].

**Table 5:** Summary of diseases and mite infestations associated with *Auricularia* spp.

Disease/Mite	Causal Organism	Affected *Auricularia* spp.	References
Slippery scar disease	*Scytalidium auriculariicola*	*A. polytricha*	[94]
	*S. lignicola*	*A. polytricha*	[85]
Cobweb disease	*Cladobotryum cubitense*	*A. polytricha*	[87]
	*Hypomyces cornea*	*A. cornea*	[82]
	*H. mycophilus*	*A. cornea* and *A. heimuer*	[83,84,88]
Brown rot disease	*Trichoderma pleuroticola*	*A. cornea* and *A. auricula*	[80,86]
Mite infestation	*Luciaphorus auriculariae*	*A. polytricha*	[90]
	*L. perniciosus*	*A. polytricha* and *A. auricula*	[91]
	*Tyrophagus putrescentiae*	*A. polytricha*	[92]

**Figure 8:**
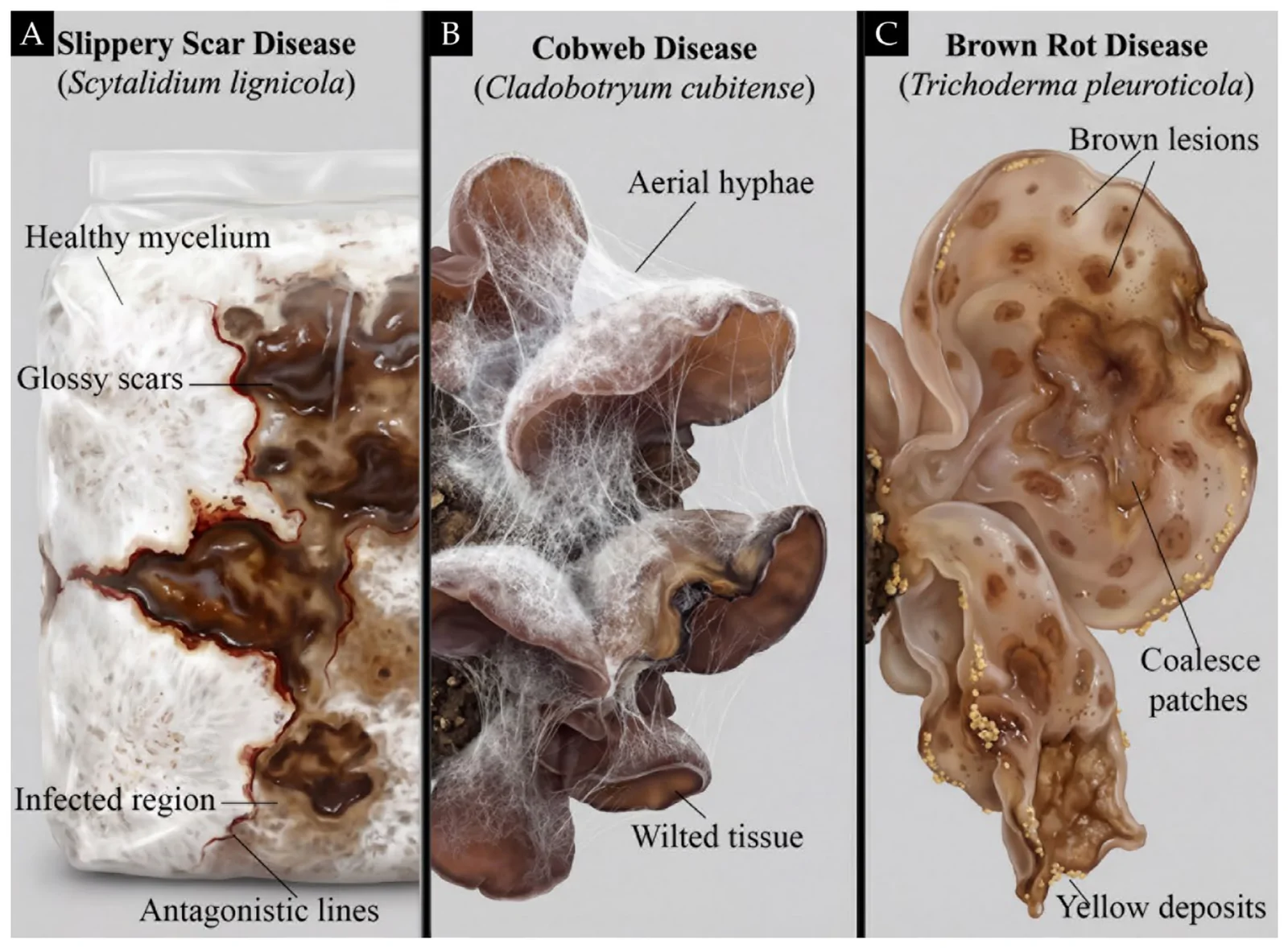
Comparative symptomatology of three major fungal diseases affecting cultivated *Auricularia* spp. Figure (**A**) shows slippery scar disease (*Scytalidium lignicola*) on colonized substrate bags, characterized by slippery, glossy, dark-brown scars on the surface of infected mycelia of *A. polytricha*. (**B**) Cobweb disease (*Cladobotryum cubitense*) appears as spiderweb-like aerial hyphae that spread over and cover the entire fruiting body, while (**C**) brown rot caused by *Trichoderma pleuroticola* is characterized by brown lesions and wilting of the fruiting body, with yellowish deposits.

In order to have effective disease management, an initial disease diagnosis is important. Therefore, a clear understanding of the characteristic symptoms associated with pathogenic fungi is essential for appropriate management. For example, slippery scar disease primarily affects *Auricularia* mycelia during active growth or after the mycelium has colonized the entire cultivation bag, rather than the fruiting bodies. The disease is characterized by slippery, glossy, dark-brown scars that appear on the surface of infected mushroom bags. A red-brown antagonistic line with an irregular margin develops between the infected and healthy mycelia. The infected scar spreads rapidly and can eventually cover the entire cultivation bag, thereby inhibiting the growth and development of *Auricularia* mycelia [85] (Figure 8). In contrast, cobweb disease primarily affects the fruiting bodies and is characterized by white, cottony or spiderweb-like mycelia. The infection initially develops as flocculent hyphae on the basal or surface areas of the fruiting bodies and rapidly spreads along the outer margins, with the hyphae developing into spiderweb-like aerial mycelia that eventually cover the entire fruiting body. This infection interferes with normal fruiting body development and causes the fruiting bodies to wilt and rot [82,83,84,88]. Brown rot disease symptoms are primarily found in newly unfolded fruiting bodies. Lesions are characterized by brown, putrefied, and foul-smelling tissue, and the fruiting bodies gradually wilt. Naturally air-dried fruiting bodies can be observed as dark brown with yellow deposits at the edges [80].

The control of pests and diseases in mushroom cultivation has traditionally relied on the application of synthetic pesticides and fungicides because of their rapid and generally effective action. Several fungicides, including difenoconazole-azoxystrobin, fluazinam-cyazofamid, iprodione, triadimefon, and trifloxystrobin-tebuconazole, have been reported as potential options for disease control. Xie et al. [82] reported that the prochloraz manganese chloride complex is the most suitable for controlling cobweb disease other than the aforesaid fungicides. However, the use of synthetic pesticides in mushroom production should be carefully considered because of possible environmental and food-safety risks. A survey of edible mushrooms collected from Czech markets detected 21 pesticide residues, metabolites, or synergists, with bifenthrin and permethrin detected in dried *A. auricula-judae* [95]. In addition, integrated approaches combining preventive, physical, biological, and chemical methods may help reduce reliance on chemical pesticides and limit fungal contamination [95,96].

In order to minimize disease incidence and limit pathogens, several disease management practices can be conducted during *Auricularia* cultivation. Optimizing environmental conditions, such as temperature, relative humidity, ventilation, and substrate composition, is critical for maintaining favorable mushroom growth while limiting pathogen development. Although high humidity favors *Auricularia* growth, the high-temperature and high-humidity conditions for cultivation can also facilitate the propagation and spread of pathogenic microorganisms, increasing the risk of diseases and pests [81]. Regular removal of infected fruiting bodies, thorough sanitation of cultivation equipment and facilities, and the use of pathogen-free or properly sterilized substrates are also essential for minimizing pathogen infection or contamination.

In a preliminary study, Qu et al. [97] reported that changes in bacterial communities associated with mushroom hosts can inhibit mites, naturally avoiding pest attacks on *Auricularia*. However, these studies needed to be more validated, and how the findings can be used for commercial cultivation needed to be further studied. Biological control measurements are not well-known for disease management associated with *Auricularia* cultivation. However, a handful of studies have recognized the potential application of microbes; e.g., Kim et al. [86] attempted to control *Trichoderma* spp. and *Penicillium* spp., which pose a significant problem for the substrates of black *Auricularia*. They identified that *Streptomyces* sp. (isolate: JC203-3) effectively controls those pathogens. Overall, integrated pest management (IPM) strategies combine chemical, biological, and cultural controls. Recent advances include the use of molecular diagnostics for early pathogen detection, the deployment of resistant cultivars, and the manipulation of the cultivation microbiome to suppress disease [86,98].

## 6. Nutritional and Health-Promoting Properties

The nutritional and health-promoting properties of *Auricularia* spp. are associated with their distinctive chemical composition, which is characterized by high content of complex polysaccharides, dietary fiber, proteins, minerals, and other low-molecular-weight bioactive constituents [9,99,100]. However, the nutritional composition of *Auricularia* varies considerably depending on species, strains, cultivation substrates, developmental stages, and postharvest processing conditions.

### 6.1. Nutritional Composition and Health-Related Nutritional Functions

The fruiting bodies of *Auricularia* spp. contain substantial amounts of carbohydrates, with polysaccharides representing one of the major components of dry matter. Fresh fruiting bodies have a high moisture content, whereas dried products are nutritionally dense, particularly in dietary fiber and minerals [8,99,100]. Protein is also an important nutritional component of *Auricularia* mushrooms, although its content varies depending on species, cultivation conditions, substrate, and analytical methods. In *A. auricula*, protein contents of approximately 10.0–16.2 g/100 g have been reported, while amino acid analyses have demonstrated the content of several essential amino acids, including isoleucine, leucine, lysine, methionine, phenylalanine, threonine, and valine [101]. Dietary fiber is another important nutritional property. In *Auricularia* and other edible mushrooms, dietary fiber is primarily a component of structural cell-wall polysaccharides, particularly β-glucans and chitin, along with other heteropolysaccharides such as glucuronoxylomannan and pectic polysaccharides [8,102,103,104]. These polysaccharide components contribute to the physicochemical and functional properties of the mushroom and have been investigated for their potential roles in gastrointestinal and metabolic health [8,104]. Carbohydrates are major components of *Auricularia* mushrooms, occurring mainly as complex polysaccharides. In *A. auricula-judae*, total carbohydrate contents of approximately 62–75% dry weight have been reported [103]. In *Auricularia heimuer*, carbohydrates are mostly composed of mannose and glucose, whereas arabinose, fucose, galactose, rhamnose, and xylose comprise approximately 10% of the total carbohydrate component [104]. *Auricularia* mushrooms also contain several vitamins and vitamin-related compounds that promote their nutritional value [103]. The fruiting bodies of *Auricularia* spp. contain ergosterol, which can be converted to vitamin D_2_ through ultraviolet (UV) irradiation [103,105]. An ergosterol content of approximately 0.1 g/100 g has been reported in *Auricularia* [103]. In addition, *Auricularia* mushrooms contain a range of essential minerals, with potassium generally being the main mineral, along with calcium, iron, magnesium, phosphorus, zinc, and other trace elements [104,106,107]. In *A. heimuer*, potassium is the most abundant mineral, accounting for approximately 70% of the total mineral content, followed by calcium, copper and magnesium, while sodium, iron and zinc were present at lower contents [104].

### 6.2. Bioactive Compounds, Structure–Activity Relationships and Health-Promoting Properties

Beyond their nutritional value, *Auricularia* species have attracted significant scientific attention because of their potential medicinal and health-promoting properties, which have been associated with a diverse range of bioactive compounds, particularly polysaccharides. Experimental studies have reported that *Auricularia* polysaccharides exhibit a broad range of biological activities, including antioxidant, immunomodulatory, hypoglycemic, hypolipidemic, anticoagulant, antithrombotic, hepatoprotective, and antitumor activities [10,103,108,109]. These biological activities are associated with structural characteristics of the polysaccharides, including molecular weight, monosaccharide unit, glycosidic linkages, degree of branching, and chain conformation, which can influence their biological functions [10,103,109]. In particular, *Auricularia* polysaccharides have been reported to modulate macrophage activity, cytokine production, phagocytic responses, and intracellular immune-signaling pathways, including the mitogen-activated protein kinase (MAPK) and nuclear factor-kappa B (NF-κB) pathways, suggesting that interactions between structurally specific polysaccharides and pattern-recognition receptors may contribute to their immunomodulatory effects [110,111].

Antioxidant activity is another well-documented medicinal property of *Auricularia* mushrooms. In vitro studies have demonstrated that *Auricularia* extracts, which are rich in polysaccharides and phenolic compounds, exhibit effective free radical scavenging activity in assays such as 2,2-diphenyl-1-picrylhydrazyl (DPPH), 2,2′-azino-bis (3-ethylbenzothiazoline-6-sulfonic acid) (ABTS), and ferric-reducing antioxidant power (FRAP) assays [10,103]. In addition to polysaccharides, *Auricularia* spp. contain phenolic compounds, melanin, proteins, sterols, flavonoids, and other secondary metabolites that may contribute to their biological activities [10,103]. Among these compounds, phenolic compounds and melanin have attracted particular attention because of their potential antioxidant activity. Melanin from *A. auricula* has also been investigated for its potential antibiofilm activity [10,112].

## 7. Future Prospects

Wood ear mushroom cultivation has advanced substantially from traditional log cultivation to intensive substrate-based production techniques. However, increasing demand for sustainable foods, climate-resilient agriculture, and high-value functional products presents new opportunities for further innovation in *Auricularia* cultivation. Future research should focus on developing high-yielding, disease-resistant, and climate-adapted *Auricularia* strains through integrated breeding programs, genomic selection, and molecular-assisted approaches. Advances in whole-genome sequencing, transcriptomics, metabolomics, and gene-editing technologies are expected to improve understanding of the genetic mechanisms regulating mycelial growth, fruiting body development, stress tolerance, and biosynthesis of bioactive compounds. The development of sustainable cultivation systems should also receive greater attention. Future substrate research should expand across conventional sawdust-based formulations and incorporate locally available agricultural and forestry residues, food-processing byproducts, and other lignocellulosic wastes into a circular bioeconomy concept. Optimizing substrate formulations through nutrient balancing, nutritional supplementation, and the use of beneficial microbial combinations could further improve biological efficiency, mushroom quality, and production consistency while reducing cultivation costs and environmental impacts. In addition, integrating *Auricularia* cultivation with agroforestry, crop-livestock practices, and organic waste recycling also offers considerable potential to enhance resource-use efficiency, reduce biomass burning, and contribute to carbon sequestration and sustainable rural development.

Technological innovation will play an increasingly important role in commercial *Auricularia* production. The adoption of standardized cultivation practices, Internet of Things (IoT)-based environmental monitoring, artificial intelligence-assisted climate control, and mechanized harvesting could significantly improve production efficiency, labor utilization, and product quality. In parallel, rapid molecular diagnostic tools, biosensors, and predictive disease models could facilitate the early detection and effective management of emerging pathogens and pests while reducing dependence on synthetic pesticides.

Despite considerable progress, several important knowledge gaps remain. More importantly, the current literature lacks systematic multivariate frameworks that integrate cultivation parameters across the entire production system. Future studies should focus on integrated approaches that evaluate the complex interactions among species or strain selection, substrate formulation, spawn rate, incubation microclimate, dynamic CO_2_ thresholds, air-exchange rates, and photoperiod cycles. Quantifying the combined effects of these factors on total production-cycle duration, flush frequency, total yield, and biological efficiency will be important for developing optimal yield roles and standardized cultivation protocols suitable for different climatic regions. Furthermore, long-term economic assessments, life cycle assessments (LCA), carbon footprint analyses, and techno-economic evaluations are needed to quantify the sustainability and commercial feasibility of different production systems.

Overall, future advancements in *Auricularia* cultivation will depend on multidisciplinary collaboration integrating fungal biology, biotechnology, agricultural engineering, environmental science, and digital agriculture. Such integrated approaches will not only improve productivity, profitability, and product quality but also strengthen the role of *Auricularia* in sustainable food systems, agricultural waste valorization, climate change mitigation, and the global bioeconomy.

## 8. Conclusions

During the last few decades, *Auricularia* cultivation has shown a significant change from traditional log-based cultivation to modern substrate-based techniques. Nevertheless, it remains uncertain whether widely adopted cultivation practices can consistently achieve the requirements for efficiency, product quality, and sustainability. Successful *Auricularia* cultivation depends on suitable lignocellulosic substrates and spawn types, as well as optimized environmental conditions for mycelial growth and fruiting body development, including pH, temperature, nutrient availability, humidity, light, and gas exchange. Substrate formulation is a critical factor of mushroom yield and biological efficiency. Substrates with very high or unbalanced carbon-to-nitrogen (C/N) ratios may limit mycelial growth and fruiting performance in *Auricularia*. The utilization of agricultural and forestry residues as cultivation substrates not only enhances mushroom productivity but also serves to recycle biomass waste, promote environmental sustainability, and support the development of circular bioeconomy systems. In addition, improvements in spawn types, including liquid and stick spawn, provide promising alternatives for improving inoculation efficiency and supporting large-scale commercial production. Moreover, effective postharvest handling and integrated disease and pest management are essential for maintaining *Auricularia* quality, safety, and productivity. Beyond the economic value, *Auricularia* mushrooms represent valuable functional foods due to their favorable nutritional composition and diverse bioactive compounds.

Nevertheless, challenges remain in standardizing cultivation methods and protocols across species and geographical regions, and in understanding interactions among strain genetics, substrate composition, environmental conditions, and the limited associated microbes. Future research should focus on multidisciplinary approaches integrating fungal biology, genomics, biotechnology, precision agriculture, and digital cultivation technologies to develop high-yielding, disease-resistant, and climate-resilient strains and cultivation systems. Such advances could further strengthen the role of *Auricularia* species in sustainable agriculture, functional food production, agricultural waste valorization, and the expanding global bioeconomy, while contributing to the achievement of the Sustainable Development Goals (SDGs).

## Figures and Tables

**Table 2:** Comparative performance of different spawn types and substrates used for the cultivation of *Auricularia* species.

Spawn Types	*Auricularia* Species	Spawn Substrate/Material	Inoculation Rate per Bag	Fruiting Conditions (Temperature and Humidity)	Incubation Period (Day)	Production (Average Yield, g)	BE (%)	References
Grain spawn	*A. cornea*	wheat grain	2%/2.5 kg	22–24 °C 64−71%	17–21	353.0–908.6	41.5–106.9	[65]
		rye grain	1%/1 kg	28 °C 85–95%	52.9	57.6	16.5	[66]
		sorghum	NA	25 °C 75–85%	NA	19.4–31.0	NA	[17]
	*A. polytricha*	cracked corn seeds	40 g	30–32 °C	29.0–35.0	14.2–75.6	2.8–15.1	[53]
		sorghum	NA	NA	30.3–38.3	29.6–254.0	3.6–30.8	[62]
		sorghum	5 g/800 g	28–30 °C	31.4	NA	NA	[67]
	*A. thailandica*	rye grain	NA	28 °C 85–95%	14.2	60.8	17.1	[7]
Sawdust spawn	*A. cornea*	beech sawdust and wheat bran	10 g/825 g	15–20 °C 85–90%	19.7–41.5	56.7–131.7	23.1− 53.1	[52]
		coarse oak sawdust, fine oak sawdust, and maize straw	10 g/1 kg	25 °C 30–40%	28.3–33. 7	196.9–457.3	67.1–130.3	[54]
	*A. polytricha*	rubber sawdust	5 g/800 g	28–30 °C	35.8	NA	NA	[67]
Liquid spawn	*A. auricula*	potato, wheat bran, glucose, peptone, brown sugar, MgSO_4_, and KH_2_PO_4_	15 mL/700 g	22 °C 90–95%	40–50 *	19.0–25.0 *	6.0–8.0 *	[58]
	*A. polytricha*	potato dextrose broth	5 mL/800 g	28–30 °C	31.8	NA	NA	[67]
Stick spawn	*A. heimuer*	*Populus* branches + substrate (sawdust-based) + 10 mL of liquid spawn	3 branches/1250 g	20–22 °C 80–90%	27.3–30.0	157.7–350.9	34.0–70.0	[59]
	*A. polytricha*	giant mimosa stalk	1 piece/800 g	28–30 °C	26.8	NA	NA	[67]

Note: NA = available/not reported, * = approximate values, and BE = biological efficiency. Chemical compounds are abbreviated as follows: MgSO_4_ = magnesium sulfate and KH_2_PO_4_ = potassium dihydrogen phosphate.

**Table 3:** Summary of optimal nutritional and environmental conditions for mycelial growth of *Auricularia* species.

Species	Culture Media	Carbon Source	Nitrogen Source	Temperature (°C)	pH	References
*A. auricula-judae*	PDA and MCMA	mannose	malt extract	25–30	6.0–9.0	[47]
*A. cornea*	rice bran sucrose agar	sucrose	-	25	5.0–7.0	[17]
*A. cornea* (white and brown strain)	MCM broth	-	-	30	6.0–7.0	[68]
*A. cornea*	PDA	mannitol	yeast extract	30	7.0	[69]
*A. delicata*	PDA	rhamnose	beef extract and peptone	28	5.5–6.0	[70]
*A. polytricha*	CWG	-	-	-	-	[62]
*A. polytricha*	YM medium	sucrose	yeast extract	25	5.0	[71]
*A. polytricha*	PSA	sucrose	-	32	6.0–7.0	[53]

**Table 4:** Environmental requirements for fruiting body development of *Auricularia* species.

Species	Temperature (°C)	RH (%)	Light (Lux)	References
*A. auricula*	18–30	90–95	12 h light/dark cycle	[41,48,55,56,58]
*A. cornea*	22–30	75–90	2000 lux, 12 h light/dark cycle	[15,17,52,54]
*A. delicata*	17–28	60–95	12 h light/dark cycle; light intensity 33–4580 lux	[70,72]
*A. heimuer*	20–22	80–90	Not reported	[59]
*A. polytricha*	23–32	80–96	179–200 lux (cool white fluorescent, 24 h/day)	[13,16,51,61,73,74]

## Data Availability

All data are contained within the article. The authors declared that generative AI was not used in the writing of this manuscript. However, generative AI (muset.ai, version: Canary 0.22.8) was used to assist in the creation of Figure 2 and Figure 8. The authors generated the initial image using a custom prompt. Subsequently, the images were manually edited, corrected, and finalized by the authors using Adobe Photoshop (version: 22.0.0) to ensure accurate representation of the content.
